# Pediatric intussusception due to basidiobolomycosis: a case report and literature review

**DOI:** 10.1186/s12887-022-03495-9

**Published:** 2022-07-20

**Authors:** Sameera Mohmmed Aljehani, Tasneem Ibraheem D. Zaidan, Noora Obaid AlHarbi, Bader Hassan Allahyani, Baha Ridah Zouaoui, Reham Hamed Alsaidalan, Saud Mohammed Aljohani

**Affiliations:** 1 Department of Pediatric-Infectious Diseases, King Abdulaziz Hospital, Jeddah, Saudi Arabia; 2 Department of Ophthalmology, Imam Abdulrahman Bin Faisal University, Al-Raka, PO Box 7191, Dammam, 11462 Kingdom of Saudi Arabia

**Keywords:** Basidiobolomycosis, Gastrointestinal, Intussusception, Pediatric, Voriconazole, Surgery, Soil

## Abstract

**Background:**

Pediatric gastrointestinal basidiobolomycosis is an unusual fungal infection caused by *Basidiobolus ranarum*, an environmental saprophyte found worldwide. Typically, basidiobolomycosis presents as a subcutaneous infection or soft tissue tumor-like lesion, and rarely involves the gastrointestinal tract. Gastrointestinal basidiobolomycosis is most common in young infants. It has no definitive clinical presentation, and almost all cases are misdiagnosed during the initial presentation.

**Case presentation:**

We report the case of a 4-year-old Saudi boy who presented to the emergency department with abdominal pain, nausea, vomiting, and weight loss. Ultrasonography revealed a target sign. Based on the ultrasonography findings, surgery was performed, which revealed the presence of intussusception. Eventually, the patient was diagnosed with intussusception secondary to intra-abdominal basidiobolomycosis based on the histological findings. The patient was readmitted and intravenous voriconazole therapy was initiated. One week after the second admission, the patient developed abdominal pain, nausea, vomiting, inability to hold down food, and constipation. Computed tomography of the abdomen was suggestive of small bowel obstruction, which was managed conservatively. The patient responded well and was subsequently discharged with a prescription of oral voriconazole.

**Conclusions:**

This case reveals that gastrointestinal basidiobolomycosis can cause intussusception. This report will inform clinicians of the importance of considering gastrointestinal basidiobolomycosis in the differential diagnosis of chronic abdominal pain in children, even in the absence of fever or a clinically obvious abdominal mass, especially in countries such as Saudi Arabia, where cases have been reported.

## Background

*Basidiobolus* is a fungus belonging to the zygomycetes class found in great abundance in the environment [1, 2]. Even though it is more common in the tropical and subtropical regions of the globe, some studies reported that this fungus was isolated in places where the disease has not been reported, such as the northeastern parts of the United States and Australia. The prevalence of basidiobolomycosis is higher in humans living in tropical regions and is even endemic to countries such as India and Uganda [3]. *Basidiobolus ranarum* was first isolated from frogs in 1886. Although most of its life cycle is spent in the intestines of agamid lizards, the spores and mycelia are found in excreta, enabling transmission to other organisms. *B. ranarum* has also been isolated in insects, dogs, bats, horses, and humans. The first reported case of this disease was a subcutaneous infection in 1956 [1, 2]. A review conducted in 2019 stated that fewer than 80 cases had been reported by 2019, of which 17 were reported between 2009 and 2014. It has been speculated that the incidence has increased in recent years because of the general increase in the number of immunocompromised people worldwide [3].

Herein, we report a case of 4-year-old Saudi boy, who presented to the emergency department with abdominal pain, nausea, vomiting, and weight loss. He was eventually diagnosed with intussusception secondary to intra-abdominal basidiobolomycosis.

Abdominal mass and eosinophilia are the most common presentations of gastrointestinal basidiobolomycosis (GIB), while intussusception is an unusual manifestation. We found only one case report of GIB from Iran [4], making this study only the second case report of basidiobolomycosis with intussusception.

## Case presentation

A 4-year-old boy living in the southern region of Saudi Arabia, who did not have any history of health problems, was not on any medication, and had no contact with animals such as dogs, frogs, and lizards, presented to the emergency department of King Abdul-Aziz Hospital in Jeddah with a history of persistent abdominal pain (since 1 month). Upon onset, the pain was colicky in nature, and was associated with persistent nausea, non-bilious vomiting with food content, and weight loss, with a negative history of fever, changes in bowel habits such as constipation, diarrhea or red currant jelly stool, jaundice, dysphagia, pica or urinary changes. The family reported that they had sought medical care three times, but each time the patient had been given analgesics (acetaminophen and nonsteroidal anti-inflammatory drugs), which only provided temporary relief. The pain was generalized, non-specific, and intermittent, and had worsened progressively during the past 10 days. The pain interfered with sleep and daily activities.

**Fig. 1:**
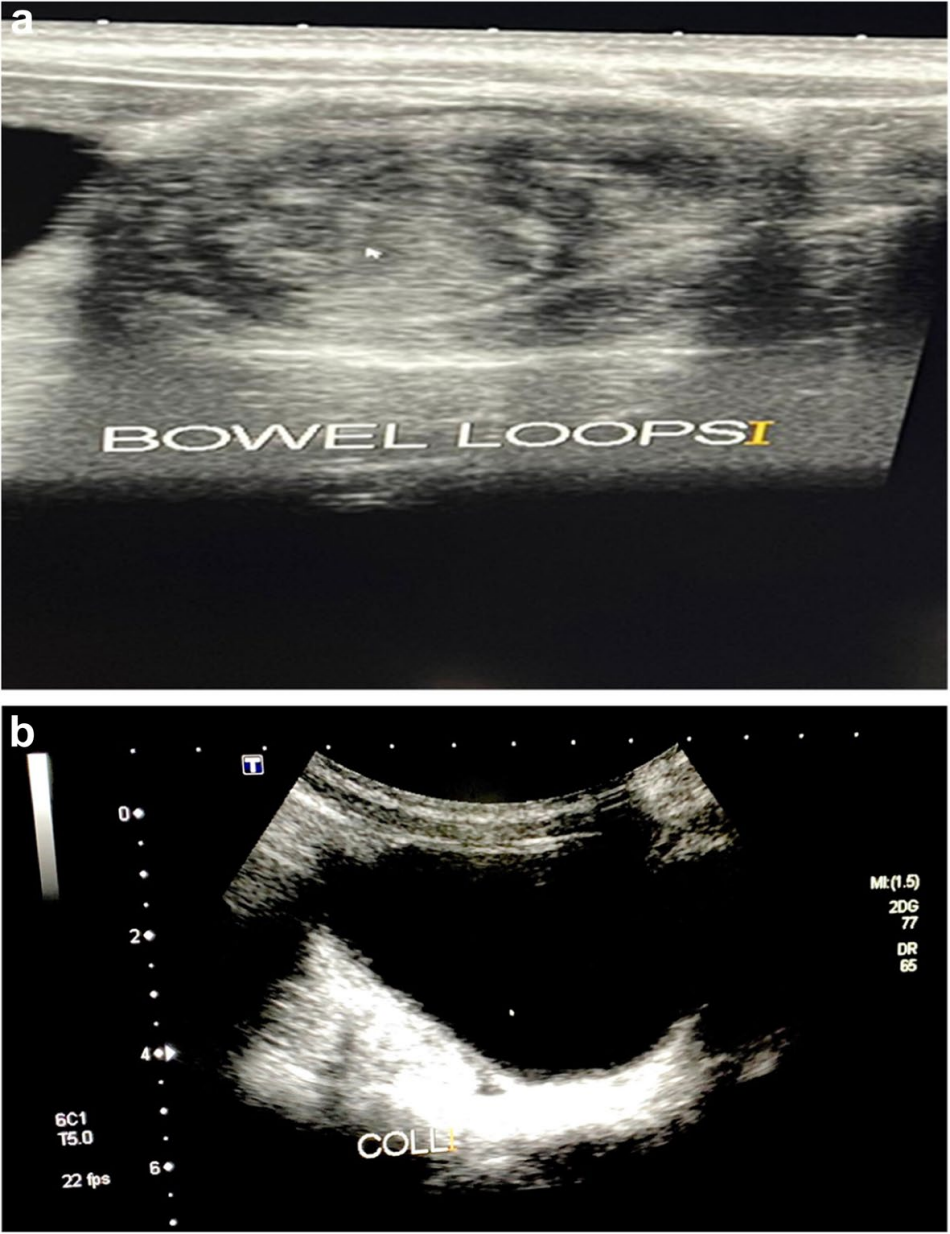
Transverse sonography of the patient’s abdomen performed during the emergency department visit (**A**) Ultrasonography showed a swirled pattern of alternating hyper-echogenicity and hypo-echogenicity in the right lumbar region. This represents the alternating layers of the mucosa, muscularis, and serosa seen in intussusception giving it a “target” appearance. **B** Moderate amount of free intraperitoneal fluid

On examination in the emergency department, the patient was conscious, alert, cachectic, and in pain. His vital signs on examination were as follows: pulse, 110 beats per min; blood pressure, 95/64 mmHg; oxygen saturation, 99% at room air; and temperature, 37.3 °C. Physical examination revealed a soft and lax abdomen, without rigidity or guarding, no palpable abdominal mass, and no localized tenderness. The initial laboratory workup revealed the following: white blood cell (WBC) count, 10,500 cells/μL with 10.5% eosinophils; hemoglobin level, 11.6 g/dL; platelet count, 362 × 10^3^/μL; normal electrolyte profile (Na, K, Cl, Mg, P); C-reactive protein (CRP) level, 3.4 mg/L; and erythrocyte sedimentation rate (ESR), 30 mm/hr. Abdominal ultrasonography revealed matted bowel loops in whorled formation in the right lumbar region, resembling a target sign, with a small amount of free intraperitoneal fluid (Fig. 1A and B).

After consultation with a pediatric surgeon, emergency laparotomy was performed for suspected intussusception.

Intraoperative findings revealed ileocecal intussusception, which was reduced. After reduction, a palpable mass was identified in the ileocecal valve and cecum with enlarged lymph nodes. The other intra-abdominal organs were normal. Limited resection of the terminal ileum, ileocecal valve, and cecum up to the beginning of the ascending colon was performed with an ilio-ascending anastomosis in two layers and drainage in the paracolic gutter. The colonic biopsy specimen was sent for histopathological examination.

The postoperative course was smooth, and the patient was extubated and moved to the ward. The results of post-operative laboratory tests performed on the day after surgery were as follows: WBC count, 19,500 cells/μL with 6.8% eosinophils; hemoglobin level, 11.2 g/dL; platelet count, 579 × 10^3^/μL; and ESR, 30 mm/h. The CRP, alanine aminotransferase, and aspartate aminotransferase levels were 3.4 mg/L, 12 IU/L, and 32 IU/L, respectively; and the electrolyte profile was normal. Serological tests for hepatitis and human immunodeficiency virus were negative. The patient did not undergo an immunological work-up because it was not indicated. The patient stayed in the ward for 6 days and was discharged in good condition.

**Fig. 2:**
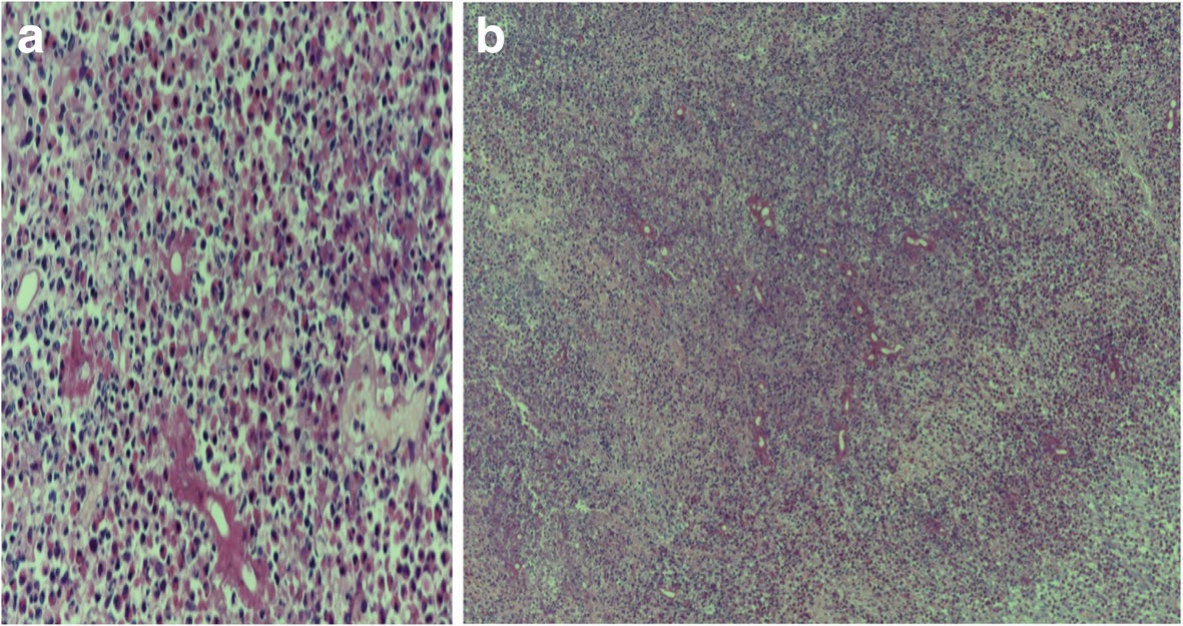
**A** Large and wide septated fungal hyphae are seen in association with nucleated basidiospores, and eosinophilic cuff aggregates condense around the fungal hyphae, forming the Splendore-Hoeppli phenomenon (hematoxylin and eosin, × 400). **B** Fungal hyphae with the Splendore-Hoeppli phenomenon: central fungal forms surrounded by a thick eosinophilic cuff (hematoxylin and eosin, × 40)

One week after discharge, histopathological examination revealed a necrotizing granuloma comprising epithelioid histocytes and multinucleated giant cells with a necrotic central area containing eosinophils. The Splendore–Hoeppli phenomenon was present, which was surrounded by oval-shaped organisms with septate hyphae (Fig. 2A and B).

We used a Nikon DS–Rio microscope with the NIS Elements imaging software.

Based on the histopathological findings, the patient was diagnosed with colonic basidiobolomycosis. Polymerase chain reaction testing was not available. Thus, the family was contacted, and the patient was readmitted for further therapy. The patient was initially administered intravenous (IV) voriconazole (7 mg/kg/dose every 12 h) with continuous monitoring of liver function. Detailed history-taking revealed that the patient played in the soil and swam in a small local lake. One week after the second admission, the patient developed abdominal pain, nausea, and vomiting, intolerance to oral intake of food, and constipation. Laboratory findings showed a WBC count of 23,000 cells/μL with mainly neutrophils, and normal hemoglobin, platelets, and electrolyte levels. Abdominal ultrasonography was requested based on a surgical consultation, and the findings were suggestive of small bowel obstruction representing adhesive bands. A decision was made to manage the bowel obstruction conservatively, and a nasogastric tube was inserted for free drainage. However, the infectious disease team suspected a healthcare-associated infection and initiated IV piperacillin-tazobactam. The patient did not show much improvement, and piperacillin-tazobactam was replaced with meropenem. Subsequently, the patient’s clinical condition improved, the abdominal pain resolved, and the patient was able to resume oral intake of food.

Two weeks after completing the course of IV meropenem, the patient was discharged with a prescription of oral voriconazole in a good general condition. Thereafter, outpatient follow-up was performed every 2 weeks for four visits, followed by monthly follow-up visits. The patient appeared to be doing well on the subsequent outpatient visits, with good oral intake of food and weight gain. The patient complied with the therapy, and the liver function test results remained normal. The patient took oral voriconazole for 1 year. During the annual follow-up, the patient showed good tolerance and compliance with the medication with no reported side-effects, and follow-up computed tomography of the abdomen showed normal findings. The patient was examined at the outpatient clinic 2 years after diagnosis and treatment and was doing well with no intestinal obstruction observed on radiological imaging.

## Discussion and conclusions

To the best of our knowledge, this is the first reported case of GIB with intussusception from Saudi Arabia. There is one previous case report of a 23-month-old immunocompetent boy living in a subtropical area in Iran [4]. Prolonged fever, presence of an abdominal mass, hepatomegaly, high ESR, and peripheral eosinophilia were strongly suggestive of GIB. Accordingly, a diagnosis of GIB was made, based on the characteristic histopathological (the Splendore-Hoeppli phenomenon) finding in the liver biopsy specimen. Exploratory laparotomy showed that several organs, including the colon, gall bladder, liver, and abdominal wall, were involved [4].

GIB is usually caused by the ingestion of water, food including fruits, or vegetables contaminated by animal fecal matter. Other proposed routes include the use of contaminated paper for cleaning the skin (e.g., toilet paper for cleaning of the skin after defecation) or implantation of the fungus following surgical procedures. *B. ranarum* is commonly found in the soil; therefore, gardeners, farmers, and landscapers are exposed to *B. ranarum* more than the rest of the population [3, 5]. Pediatric GIB is an unusual fungal infection caused by *B. ranarum* [1, 2, 6]. Typically, this organism causes subcutaneous, sinus, and nasal infection, and rarely causes a soft tissue tumor-like mass of the bowel. The classical disease has a chronic course and occurs mostly in young children as young as 1 month of age [6]. GIB was first identified in 1964 in a 4-year-old boy, and since then has been reported in several countries, including Saudi Arabia, Kuwait, and Iran [7]. GIB has no definitive clinical presentation, and the diagnosis is missed in the initial stage in almost all cases. Patients are usually misdiagnosed with other more common conditions, such as Crohn’s disease, inflammatory pseudotumor, and colorectal cancer, in the initial stage [3, 7–10].

In our case, the patient presented with severe generalized abdominal pain, nausea, vomiting, and weight loss, which were mismanaged with analgesics, and the condition was misdiagnosed by multiple healthcare providers, reflecting the difficulty in diagnosing GIB. GIB is mainly diagnosed by histological examination of the surgical specimens; thus, almost all patients develop complications before diagnosis [11].

Although the majority of the cases are diagnosed based on biopsy and histopathological findings, which includes chronic granulomas rich in eosinophils and the Splendore–Hoeppli phenomenon, the definitive diagnosis is proven by a culture, which is the gold standard, and by special staining for fungi (periodic acid-Schiff stain) [12, 13].

Furthermore, the management of this disease requires a combination of surgical and medical intervention. GIB is managed surgically using resection and/or debridement of all necrotic tissue, followed by long-term antifungal treatment (weeks to months) [14, 15]. The optimal duration of antifungal therapy for GIB is presently unknown; most experts recommend a minimum treatment duration of 6 months after surgical resection to minimize the risk of relapse. Our patient received antifungal therapy (voriconazole) for 12 months [16].

In the literature, there is a lack of consensus about the antifungal agent of choice, and some studies have reported failure of therapy with amphotericin B. Nevertheless, favorable results have been reported with posaconazole and voriconazole in GIB [15].

Finally, the route of entry of *B. ranarum* was from the soil or decaying plants, resulting in gastrointestinal disease in this patient who resides in the southern region of Saudi Arabia.

GIB is difficult to diagnose due to its rarity and non-specific clinical presentation, as it resembles several other more common diseases. Consequently, numerous cases are misdiagnosed and mistreated. We aim to broaden clinicians’ knowledge about this disease by reporting this case of an extremely unique presentation of this rare disease. Moreover, we hope for favorable outcomes when faced with such rare conditions and that our findings may pave the way for a better grasp of the treatment and management of such cases.

## Data Availability

Data sharing is not applicable to this article as no datasets were generated or analyzed during the current study.

## Abbreviations

GIB: Gastrointestinal basidiobolomycosis
WBC: White blood cell
CRP: C-reactive protein
ESR: Erythrocyte sedimentation rate
